# Rhabdomyolysis Induced by Nonstrenuous Exercise in a Patient with Graves' Disease

**DOI:** 10.1155/2014/286450

**Published:** 2014-02-11

**Authors:** Sarawut Summachiwakij, Issac Sachmechi

**Affiliations:** ^1^Department of Medicine, Icahn School of Medicine at Mount Sinai, Queens Hospital Center, Jamaica, New York, NY 11432, USA; ^2^Division of Endocrinology, Icahn School of Medicine at Mount Sinai, Queens Hospital Center, Jamaica, New York, NY 11432, USA

## Abstract

Hyperthyroidism can result in several musculoskeletal conditions such as thyrotoxic periodic paralysis, thyrotoxic myopathy, and thyroid ophthalmopathy. Rhabdomyolysis has been rarely reported to be associated with hyperthyroidism. We describe a 33-year-old man who presented with bilateral thigh pain and dark brown urine after regular squatting. He had a past medical history of hyperthyroidism but stopped taking it 2 months prior to admission. He was found to have rhabdomyolysis, myoglobinuria, and thyrotoxicosis. Presence of thyroid-stimulating immunoglobulins (TSI) and high radioiodine uptake confirmed a diagnosis of Graves' disease. He received aggressive fluid resuscitation and sodium bicarbonate intravenously along with monitoring fluid and electrolyte. Methimazole was also resumed. The patient responded to treatment and rhabdomyolysis gradually resolved. Therefore, nonstrenuous exercise can potentially induce rhabdomyolysis in patients with hyperthyroidism. Although hyperthyroidism is not widely recognized as a cause of rhabdomyolysis, it should be considered in the differential diagnosis of rhabdomyolysis.

## 1. Introduction

Rhabdomyolysis is characterized by muscle necrosis resulting in the release of muscle cell content into the circulation. This condition has been occasionally associated with strenuous exercise, hyperthermia, certain infections, and metabolic abnormalities such as diabetic coma, severe electrolyte disturbances, and hypothyroidism [1]. In the English literature, hyperthyroidism has been rarely reported to be associated with rhabdomyolysis [1–6]. We have described the case with hyperthyroidism who developed rhabdomyolysis after nonstrenuous exercise.

## 2. Case Presentation

A 33-year-old male presented with bilateral thigh pain after regular squatting. The patient often squatted for exercise and never developed rhabdomyolysis. On the following day, the patient developed severe bilateral pain and stiffness in both thighs. He took Tylenol for pain and denied using any steroid or illicit drugs. He noticed dark brown-colored urine which urged him to come to the hospital. There was no history of fever, cough, sore throat, headache, blurred vision, palpitations, diarrhea, or abdominal pain. The patient also had a past medical history of hyperthyroidism and was taking methimazole which he stopped 2 months prior to admission. On physical examination, the patient was alert and oriented in no acute distress. His blood pressure was 137/77 mmHg and pulse was 100 beats per minute, and he was afebrile. His eyes showed no lid lag or exophthalmos. An examination of neck revealed mild thyroid enlargement on swallowing but not tender. No thyroid bruit was heard. Heart and chest examinations were normal. Examination of thighs showed mild swelling and tenderness. Neurological examination showed no hand tremor, normal muscle power, and deep tendon reflex 2+ all. The remainder of the physical examination was unremarkable. A chest X-ray and an ECG were normal.

The laboratory findings revealed the following values: blood urea nitrogen 10 mg/dL (8–22 mg/dL), creatinine 0.8 mg/dL (0.4–1.6 mg/dL), sodium 140 mg/dL (136–146 mg/dL), potassium 4.4 mg/dL (3.5–5.3 mg/dL), carbon dioxide 29 mg/dL (23–32 mg/dL), chloride 103 mg/dL (98–110 mg/dL), phosphate 4.6 (2.4–4.1 mg/dL), magnesium 1.97 (1.7–2.2 mg/dL), glucose 83 mg/dL (74–115 mg/dL), a hemoglobin 16.2 g/dL (13.5–17.5 g/dL), a white blood cell count 9.3 K/mcL (4.5–11 K/mcL) with a differential of 72.9 percent neutrophil, 17.7 percent lymphocyte, and 8.2 percent monocyte, platelet 217 K/mcL (130–400 K/mcL), CK 98407 IU/L (26–189 IU/L), thyroid stimulating hormone 0.01 mIU/mL (0.34–5.6 mIU/mL), free T4 1.98 ng/dL (0.58–1.64 ng/dL), T3 176.5 ng/dL (87–178 ng/dL), alkaline phosphatase 66 U/L (30–115 U/L), AST 993 U/L (2–40 U/L), ALT 228 U/L (2–50 U/L), LDH 2330 U/L (90–225 U/L), albumin 2.8 g/dL (3.5–5 g/dL), total bilirubin 1.63 mg/dL (normal < 1.5 mg/dL), and direct bilirubin 0.39 mg/dL (normal < 0.3 mg/dL). Urinalysis showed a specific gravity of 1.021, blood large, ketone trace, protein 300 mg/gL, red blood cell 0–4, white blood cell 0–4, urine myoglobin 36 mcg/L (normal < 28), and serum myoglobin 2250 mcg/L (normal 0–60). Urine toxicology was negative. TSH receptor antibody 48% (normal < 10%), antithyroglobulin antibody, and anti-thyroid peroxidase antibody were negative.

The patient was diagnosed with rhabdomyolysis and was treated with aggressive intravenous fluid and sodium bicarbonate drip. Urine output and electrolyte were closely monitored. He was started on methimazole for hyperthyroidism. His symptom significantly improved and serum creatinine kinase level also gradually decreased without electrolyte imbalance or acute kidney injury. The patient was discharged home with stable condition and had a radioactive iodine uptake done which showed high radioactive iodine uptake with homogenous activity consistent with Graves' disease.

## 3. Discussion

Musculoskeletal manifestations of hyperthyroidism include thyrotoxic periodic paralysis, thyrotoxic myopathy, and less commonly thyroid ophthalmopathy [5]. Rhabdomyolysis known to be more frequently occurred in hypothyroidism has been rarely reported to be associated with hyperthyroidism.

Theoretically, hyperthyroidism can cause rhabdomyolysis by increasing cellular metabolism and also decreasing muscle energy stores, both of which result in cellular damage [3]. Disturbance in the level of skeletal muscle carnitine may play a role in the pathogenesis. Carnitine which is substance necessary for the production of energy was significantly reduced in skeletal muscle of hyperthyroid patients. The mechanism by which carnitine becomes depleted might be due to an increased esterification and urinary excretion of carnitine [7].

The summaries of cases of rhabdomyolysis associated with hyperthyroidism are detailed in Table 1. Three cases of rhabdomyolysis occurred in the setting of thyroid storm [2, 3, 5]. One of these cases developed heart failure and liver failure and died of disseminated intravascular coagulation and renal failure [3]. The other cases of rhabdomyolysis did not meet the criteria for thyroid storm [1, 4, 6] and one case was triggered by a regular exercise session [6]. Therefore, hyperthyroidism should be considered in the differential diagnosis of rhabdomyolysis. Furthermore, in patients who developed rhabdomyolysis after nonstrenuous exercise hyperthyroidism should be ruled out.

## Figures and Tables

**Table 1:** Cases of rhabdomyolysis associated with hyperthyroidism published in the medical literature.

Case number	Age/sex	Past medical history	Presenting symptoms	Hyperthyroid state	Possible triggering factors of rhabdomyolysis	Complication/outcome	Reference
1	26/F	NA	Altered mental status	Thyroid storm	Infection	Acute renal failure/resolved	[2]
2	50/M	Graves' disease	Altered mental status	Thyroid storm	NA	Acute renal failure, heart failure, liver failure, DIC/expired	[3]
3	20/M	NA	Weakness and muscle cramp	Thyrotoxicosis	NA	Resolved	[4]
4	53/F	NA	Altered mental status	Thyroid storm	Infection	Acute renal failure/resolved	[5]
5	26/F	Hypertension	Blurred vision, headache	Thyrotoxicosis	NA	Resolved	[1]
6	23/M	NA	Flank pain	Thyrotoxicosis	Exercise	Resolved	[6]
7	33/M	Hyperthy roidism	Thigh pain	Thyrotoxicosis	Exercise	Resolved	Present case
